# Glucose Toxicity: a case report

**DOI:** 10.1186/1757-1626-1-295

**Published:** 2008-11-03

**Authors:** Stanley S Lefkowitz, Doris L Lefkowitz, Daniel Le, Greg Charles, James Mone

**Affiliations:** 1 Department of Molecular Genetics and Microbiology, University of Texas, Austin, TX, USA; 2 Daniel Le, 351 Hospital Rd, Newport Beach, CA, USA; 3 Greg Charles, Computer Associates, One CA Plaza, Islandia, NY, USA; 4 James Mone, Department of Biology, Millersville University, Millersville PA, USA

## Abstract

**Background:**

Toxicity to glucose is not a normal state, however, it has been reported that after certain individuals ingest sugar, they produce excess tumor necrosis factor.

**Case report:**

An other wise healthy 47 year old male developed a severe sensitivity to foods containing sugar over a 12 year period. After eating certain foods containing sugars he would develop angst, pain in his extremities, fever, etc. Onset was gradual and he has had to eliminate various foods over the years until today he is restricted to eating only certain foods without exception. In a controlled challenge with glucose, tumor necrosis factor was found to be elevated in his serum.

**Conclusion:**

Because mannose is known to block induction of tumor necrosis factor by macrophages, a regimen of mannose either prior to and/or post development of symptoms minimized these responses. A precise understanding of these events awaits further study.

## Introduction

Toxicity to glucose is not a normal state, however, it has been reported that after certain individuals ingest sugar, they produce excess tumor necrosis factor (TNF) [1]. When their monocytes were challenged *in vitro *they produced reactive oxygen species which prevailed over the induction of insulin. This was termed the "joker effect" or glucose-induced genotoxicity.

## Case presentation

A 47 year old extremely athletic male who competed in track events, which included pole vaulting, won numerous awards and all American status for his athletic prowess. Approximately 12 years ago, he noticed that when he would ingest certain foods containing sugar, he would develop a constellation of symptoms within minutes. It first started with eating melon, then frozen yogurt, and then virtually all foods containing small amounts of sugars. These symptoms included: angst, discomfort, and nausea, followed by flu-like symptoms included headache and muscle discomfort. Subsequently he would develop fever up to 103 degrees and neuropathic-like pain in the extremities. These symptoms have become more severe in recent years. His diet for the past 18 months consisted exclusively of 4 oz of beef or chicken and white rice for each meal with no exceptions. Any deviation resulted in the symptoms described above. In addition, he was taking certain medications on a regular basis, which contained lactose, and was becoming increasingly "sensitized" to these sources of sugar as well. His weight has dropped from 178 lbs to his present 148 lbs.

Since he was an extremely athletic individual and had participated in track events including running and pole vaulting etc., he found that intense physical activity could alleviate these symptoms. He noted that with repeated sprints between 50 and 100 meters with 10 repetitions, he could minimize the pain and discomfort following ingestion of sugars. Moderate exercise did little to ameliorate symptoms. He consulted with numerous physicians over a number of years. Internists, allergists, and endocrinologists afforded him little relief because all of his laboratory values were essentially normal. CAT scans, MRI's, EMG's and extensive laboratory tests were done with no abnormal findings except for a 3 fold elevation of calcium channel activity and an elevated interleukin-8 baseline titer. A presumptive diagnosis of diabetes was subsequently ruled out.

Noting the rapidity of symptoms (15 to 30 minutes) following ingestion of sugars, it suggested that cytokines such as TNF could be involved in the above syndrome. Furthermore, it has also been reported that strenuous exercise can result in the induction of interleukin 6 (IL-6) which can alleviate symptoms triggered by TNF [2]. Investigators reported that IL-6 stimulates the production of interleukin 1 receptor antagonist and stimulates interleukin10, a known TNF antagonist. Because of the known ability of mannose to block the mannose receptor on macrophages [3] resulting in decreased production of TNF, we suggested that he obtain some mannose to see if it could block the untoward response to his medication containing lactose. Mannose, either in capsule form, or placed directly on his tongue prior to each dose of medication was quite successful in alleviating symptoms, even when he could not identify the source of the sugar. Invariably, administration of mannose caused a profound reduction of his symptoms and aborted the more serious complications which would follow. He now carries a supply of mannose capsules with him wherever he goes. The amounts of mannose employed depended on the severity of the reaction. Multiple doses varying from 1 to 3 grams each (2–6 capsules) at 15 minute intervals were successful at ameliorating the symptoms.

In order to further clarify the nature of the response we suggested a challenge with a known amount of glucose followed by a rescue with mannose. Under controlled conditions, GC took approximately 200 mg of glucose, in divided doses for safety reasons, and responded within minutes with the usual symptoms. Blood samples were withdrawn every 20 minutes and stored at -70° for TNF assay by enzyme linked immunoabsorbent assay. In less than one hour after glucose ingestion, TNF titers rose from undetectable to approximately 45 pg/ml, and remained elevated for at least 20 minutes. At that time, a course of oral mannose was administered resulting in undetectable TNF at the next bleeding 20 minutes later. It should be noted that normal levels of TNF are undetectable to a few pica grams per ml serum. Because his response was essentially "dose dependent" to the level of exposure, it was felt that far less than 200 mg of glucose would have also induced TNF but because of time limitations that was not possible to evaluate.

## Discussion and conclusion

On the basis of the above, it is clear that GC developed a severe toxicity to glucose ingestion. His symptoms have become progressively worse over the years, but can be alleviated by either mannose or strenuous exercise with the former far more effective than the latter. These symptoms may be caused, at least in part, by TNF which was present after ingestion of glucose. Since the patient continues to become more sensitized to glucose and continues to lose weight, further evaluation for possible treatment is currently in progress.

## Abbreviations

TNF: Tumor necrosis factor; IL-6: interleukin 6

## Competing interests

The authors declare that they have no competing interests.

## Authors' contributions

SSl was involved in the initial drafting, all authors participated in the corrections, and revisions of the manuscript, JM was responsible for the lab work. All authors have read and approved this manuscript.

## Consent

Written informed consent was obtained from the patient for publication of this report.

## References

[B1] Bernstein LM, Vasilyev DA, Poroshina TE, Kovalenko IG (2006). Glucose-induced effects and joker function of glucose: endocrine or genotoxic prevalence?. Horm Metab Res.

[B2] Pederson BK (2006). The anti-inflammatory effect of exercise: its role in diabetes and cardiovascular disease control. Essays Biochem.

[B3] Kery V, Krepinsky JJ, Warren CD, Capek P, Stahl PD (1992). Ligand recognition by purified mannose receptor. Arch Biochem Biophys.

